# Assessment of heavy metal tolerance and biosorptive potential of *Klebsiella variicola* isolated from industrial effluents

**DOI:** 10.1186/s13568-017-0482-2

**Published:** 2017-09-29

**Authors:** Abuzar Muhammad Afzal, Muhammad Hidayat Rasool, Muhammad Waseem, Bilal Aslam

**Affiliations:** Department of Microbiology, Government College University, Faisalabad, 38000 Pakistan

**Keywords:** *Klebsiella variicola*, Heavy metal tolerance, Textile effluent, Molecular characterization, Nickel, Cobalt, Biosorption

## Abstract

Heavy metal contamination now a day is one of the major global environmental concerns. Textile effluents of Faisalabad Pakistan are heavily contaminated with heavy metals and demands to explore native microorganisms as effective bioremediation tool. Study aimed to isolate heavy metal tolerant bacteria from textile effluents of Faisalabad Pakistan and to evaluate their biosorptive potential. Out of 30 collected samples 13 isolates having metal tolerance potential against Ni and Co were screened out. Maximum tolerable concentration and multi metal resistance was determined. A native bacterial strain showing maximum tolerance to Ni and Co and multi metal resistance against Ni, Co and Cr at different levels was selected and named as Abuzar Microbiology 1 (AMIC1). Molecular characterization confirmed it as *Klebsiella variicola* which was submitted in First fungal culture bank of Pakistan (FCBP-WB-0688). ICP-OES revealed that it reduced Ni (50, 49%) and Co (71, 68.6%) after 24 and 48 h, respectively. FT-IR was used to analyze functional groups and overall nature of chemical bonds. Changes in spectra of biomass were observed after absorption of Ni and Co by *K. variicola.* SEM revealed morphological changes in bacteria in response to metal stress. Both metals affected bacterial cell wall and created pores in it. However effect of Ni was more pronounced than Co. It was concluded that *K. variicola*, a native novel strain possessed significant heavy metal tolerance and bioremediation potential against Ni and Co. It may be used in future for development of bioremediation agents to detoxify textile effluents at industrial surroundings.

## Introduction

Heavy metals are naturally occurring elements that have a relatively high atomic weight and density compared to water. Their multiple applications have resulted in wide distribution in the environment; raising concerns over their potential effects on human health and the environment. Heavy metal contamination now a day is one of the major global environmental concern and the main sources of heavy metal contamination are either natural or anthropogenic. Depending on soil pH and their specification these heavy metals can become mobile in soils and in this way, a small part of the total mass can leach to aquifer or can become bioavailable to living organisms (Hookoom and Puchooa 2013). Industrial wastewater is commonly used for irrigation in most of the developing third world countries (Bouwer 2002). Without proper treatment, release of heavy metals in effluent waste poses a menace to public health because of its persistence, biomagnifications and accumulation in food chain (Issazadeh et al. 2014). As the number of industries is being increased day by day in the modern world, with this the concentration of heavy metals is also being increased. Cadmium, chromium, mercury, lead, nickel, cobalt and copper are the heavy metals mainly found in the industrial wastewater (Smrithi and Usha 2012).

Several studies have been conducted to elaborate the effects of these heavy metals on living organisms including animals, plants and human (Chisti 2004). Heavy metals can damage the living organisms through different mechanisms such as by affecting the cell membranes, by altering the enzymes specificity, by disrupting the cellular functions and by damaging the structure of the DNA (Ozer and Pirincci 2006). In recent years, the use of microbes for removal of heavy metals has achieved great attention. Various microorganisms such as bacteria (Shuttlework and Unz 1993), yeast (Salinas and Melorza 2000), fungi (Anoop and Viraraghvan 1999), algae (Ahuja and Gupta 1999), and plants (Chen and Chen 1996) have been reported to tolerate and remove heavy metals from aqueous solutions. This situation emphasizes the need to find out more environmental friendly and cost effective treatment methods (Yasar et al. 2013).

Bioremediation is a potential cost effective solution for the remediation of heavy metal contaminated environment (Congeevaram et al. 2007). The advantages of using microbes for bio-remediation include natural occurrence, cheap production, easy availability to treat large volumes of wastewater due to rapid kinetics and high selectivity in terms of removal and recovery of specific metals. Microorganisms that are able to survive well in high concentration of heavy metals are of great interest as bioremediation agents because they can achieve different transformation and immobilization processes. Specifically, they conduct bioaccumulation based on the incorporation of metals inside the living biomass or biosorption, in which metal ions are adsorbed at the cellular surface by different mechanisms (Vijayaraghavan and Yun 2008). Indigenous organisms have the ability to adopt themselves according to the prevailing environments and could flourish under these conditions (Haq and Shakoori 2000). Variety of mechanisms has been developed by some microorganisms to deal with high concentrations of heavy metals and usually is specific to one or a few metals (Piddock 2006). Most microorganisms, posses the efflux of metal ions outside the cell, and genes for tolerance mechanisms has been found on both chromosomes and plasmids (Hookoom and Puchooa 2013). Some bacteria can use mechanisms of tolerance and detoxification of heavy metals and still produce chelating agents that bound metals and reduce their toxicity (Kavamura and Esposito 2010). Many living bacteria have been reported to reduce or to transform toxic contaminants into their less toxic forms (Solecka et al. 2012).

Faisalabad is the 3rd biggest city in Pakistan after Karachi and Lahore. It is the 2nd biggest city in the province of Punjab after Lahore, and a major industrial center. The city is also known as the “Manchester of Pakistan” (Jaffrelot 2002). The surrounding countryside, irrigated by the lower Chenab River, produces cotton, wheat, sugarcane, vegetables and fruits. Due to the heavy industrialization different types of waste is being produced by the different industries. The textile zone is playing a vital role in the export of the country but at the same time a lot of environmental pollution is being produced by this zone. It is one of the main polluters in industrial sectors in the compass of degree and the chemical composition of the discharged runoff. Inept dyeing processes often result in considerable residuals of dyes. The presence of these dyes in effluent is considered to be very problematic because of the persistent and recalcitrant nature (Yasar et al. 2013). Therefore it is need of the time to analyze these wastes for the isolation and characterization of some indigenous strains of heavy metal tolerant (HMT) bacteria and to explore their potential in bioremediation of common heavy metals founds in such effluents. Keeping in view the above, present study was conducted for the isolation, identification and molecular characterization of indigenous HMT bacteria from textile effluents and to evaluate their biosorptive potential against heavy metals.

## Materials and methods

### Sample collection and heavy metals analysis

Wastewater samples were collected from the textile effluents. Six main drains present in and around Faisalabad, Pakistan receiving the textile effluents and surrounding different textile units were selected. From each drain, five samples were taken keeping the distance of about 1000 m between two points (Baby et al. 2014). After collection, samples were processed for determination of heavy metals i.e. cobalt (Co), chromium (Cr), nickel (Ni), lead (Pb) and zinc (Zn). Samples were digested by following the protocol as previously described by Sinha and Paul (2014) and metal analysis was done using Atomic Absorption Spectrophotometer (AAS) (Hitachi Polarized Zeeman AAS, Z-8200, Japan) following the conditions described in AOAC (1990). Based on the results of metal analysis, nickel (Ni) and cobalt (Co) were selected for further study.

### Isolation of HMT bacteria

Ten fold serial dilutions of effluents were prepared in sterile distilled water up to 10^−5^ as described by Lucious et al. (2013). Isolation of Ni and Co tolerant bacteria was done through spread plate method as described by Samanta et al. (2012).

### Determination of maximum tolerable concentration (MTC)

The highest concentration that allowed visible bacterial growth after 48 to 96 h of incubation was used to select MTC of heavy metal. The increasing concentration of both heavy metals (Ni and Co) i.e..5, 1, 1.5, 2, 2.5, 3, 3.5, 4, 4.5, 5, 5.5, 6, 6.5, 7, 7.5, 8, 8.5, 9, 9.5, and 10 mM were filter sterilized first and then added into nutrient agar which was sterilized through autoclaving and cooled at room temperature for testing the MTCs of bacteria (Vashishth and Khanna 2015).

### Multi metal resistance (MMR)

Multi metal resistance of bacteria was determined by inoculating autoclaved and cooled nutrient agar medium incorporated with filtered sterilized solutions of Ni, Co and Cr in equal concentration i.e. 1:1:1 as described by Saini and Pant (2016).

### Identification of bacteria

After 48 h of incubation, colonies were selected on the basis of morphology, shape and color. All the isolates were purified by repeated streaking on nutrient agar and stored at 4 °C for further studies. Identification up to genus level was done on the basis of cultural characteristics, microscopic examination after Gram’s staining; “shape, arrangement and staining character”, and physiological/biochemical characteristics; “motility, oxidase reaction, catalase reaction, glucose utilization and fermentation tests and starch hydrolysis”. All identification tests were performed following the protocols mentioned in Bergey’s Manual of Determinative Bacteriology (1994).

### Molecular characterization

Ribotyping was done for the molecular characterization of identified HMT bacteria by amplifying 16S rRNA gene. Total genomic DNA was extracted by CTAB method (Wilson 2001). Polymerase Chain Reaction (PCR) was used for the amplification of 16S rRNA using 16S rRNA PCR primers, PA (5′-AGAGTTTGATCCTGGCTCAG-3′, and PH (5′-AAGGAGGTGATCCAGCCGCA-3′) (Zaheer et al. 2016). For ribotyping, all the isolates were grown in Luria–Bertani (LB) broth and total genomic DNA was extracted. PCR products were eluted using a gel extraction kit (Fermentas, Germany) and sent for commercial sequencing (Eurofins MWG Operon LLC, USA). After amplification, 16S rRNA sequences were compared with known sequences in the GenBank database.

### Phylogenetic analysis

The 16S rRNA gene from the pure culture sequences from the NCBI database were aligned using Clustal X (Thompson et al. 1997) and the maximum likelihood (ML)-based phylogenetic tree was constructed using MEGA (version 6) (Tamura et al. 2013). Confidence in the tree topology was evaluated using bootstrap resampling methods (1000 replications), and bootstrap values of up to 90% that demonstrated good support measures were retained.

### Effect of Ni and Co on bacterial growth

To observe the effect of Ni and Co on bacterial growth, growth curve experiment was conducted in nutrient broth. For this purpose, nutrient broth tubes with Ni (01 mM), Co (01 mM) and without Ni and Co (control) were prepared. For each bacterial isolate 100 ml medium was taken in one set consisting of eight test tubes for all three groups (i.e. two with metals and one control), autoclaved and then inoculated with 20 μl of freshly prepared inoculum. These tubes were incubated in shaking incubator at 37 °C at 100 rpm. Then after 0, 4, 8, 12, 16, 20, 24 and 28 h one tube out of eight in each group was removed and absorbance was measured at 600 nm. Growth curve was plotted by the readings obtained from the experiment and compared (Shakoori et al. 2010).

### Evaluation of biosorption potential

Biosorption potential of isolated and characterized bacterial strain named as AMIC1was determined against two metals i.e. Ni and Co. For this purpose, one set (each containing 02 glass culture bottles) having capacity of 500 ml was prepared for each metal supplemented with 200 ml of LB broth with initial metal concentration of 50 ppm for both metals. After autoclaving each set was inoculated with 02 ml of 18-h old bacterial culture having turbidity equal to 0.5 McFarland. Culture bottles were kept under constant agitation at 37 °C for 24 and 48 h then centrifuged at 14,000 rpm for 05 min and supernatants were collected and stored at − 20 °C for heavy metal analyses. Heavy metals present in solution were measured through Inductively Coupled Plasma-Optical Emission Spectroscopy (ICP-OES) (Ramyakrishna and Sudhamani 2016).

### FT-IR analysis of bacterial biomass

Fourier transform infrared spectroscopy (FT-IR) was used to analyze the functional groups and overall nature of chemical bonds in bacterial strain. Infrared spectra of the control (bacteria grown without metal stress) and tested (bacteria grown with metal stress, Ni or Co) biomass were obtained by grinding 02 mg of freeze-dried biomass with 200 mg dry potassium bromide (KBr) powder (1:100) ratio in mortar. Obtained mixture was pressed to get translucent sample discs using pressure bench press. The FT-IR analysis was performed by using PerkinElmer Spectrum Version 10.4.3. The spectral data were collected over the range of 450–4000 cm^−1^ (Ramyakrishna and Sudhamani 2016).

### Scanning electron microscopy (SEM)

Outer morphology of the bacterial cells before and after biosorption was examined using SEM (Carl Zeiss Supra 55 Gemin; German Technology, Jena, Germany). Prepared samples were placed on the sample holder (stub) with carbon tape. In order to increase the electron conduction and to improve the quality of micrographs, a conductive layer of gold was made with portable SC7620 ‘Mini’ sputter coater/glow discharge system (Quorum Technologies Ltd, Laughton, UK) (Michalak et al. 2014).

### Statistical analysis

The data was analyzed by calculating mean ± SE, analysis of variance (ANOVA), regression, co-relation and Z test was performed by using Minitab software. P value was calculated to see the significant results. Results showing P value less than 0.05 were considered as significant (P < 0.05) and whereas P value less than 0.01 as highly significant (P < 0.01).

## Results

Results of heavy metal analysis through AAS revealed the presence of Ni in the range of 0.168 ± 0.035 to 0.230 ± 0.019 mM, Co 0.128 ± 0.053 to 0.216 ± 0.008 mM, Cr 0.031 ± 0.020 to 0.098 ± 0.018 mM, Pb 0.026 ± 0.023 to 0.240 ± 0.160 mM and Zn 0.218 ± 0.068 to 0.336 ± 0.016 mM in all 30 samples. As the concentration of Ni and Co was significantly higher as compared to other heavy metals in all effluent samples therefore these two metals were selected for further study. Out of 30 samples, 13 were found to have Ni tolerant bacteria and one sample with sample code RgrDP_3_ revealed the presence of some novel bacteria which tolerated Ni up to 08 mM. This sample was selected for further purification and single colonies were obtained by repeated streaking and resultant bacterial strain was named as Abuzar Microbiology 1 (AMIC1).

**Fig. 1:**
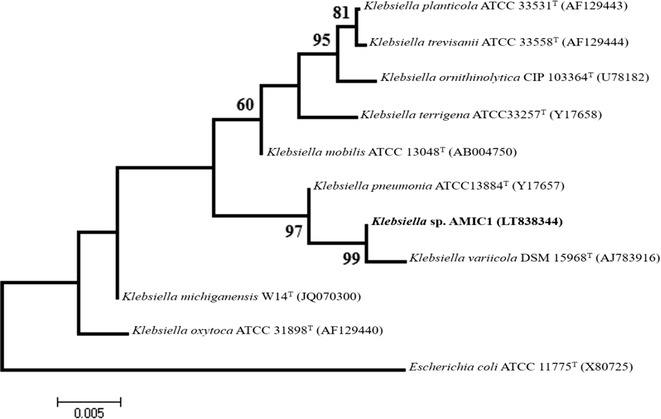
16S rRNA sequence**-**based phylogenetic tree of *Klebsiella variicola* isolated from textile effluents

Maximum tolerable concentration and multi metal resistance exhibited by AMIC1 are presented in Table 1. Initial identification up to genus level was done by using standard microbiological techniques. Gram’s staining revealed G−ve, non-motile rods (Table 2). Molecular characterization through ribotyping confirmed bacterial strain AMIC1 as *Klebsiella* spp. showing 99.79% similarity with *Klebsiella variicola* DSM 15968^T^ (AJ783916) (Type Strain: CP010523; Accession Number: LT838344). Phylogenetic tree for *K. variicola* constructed by maximum likelihood method is shown in Fig. 1. This native strain was submitted in First fungal culture bank of Pakistan (FCBP), Institute of Agricultural Sciences, University of the Punjab Lahore Pakistan under the culture accession number FCBP-WB-0688.

**Table 1:** Maximum tolerable concentration and multi metal resistance shown by AMIC1 against heavy metals

MTC of Ni	Concentration of Ni (mM)																
	1	1.5	2	2.5	3	3.5	4	4.5	5	5.5	6	6.5	7	7.5	8	8.5	9
	+	+	+	+	+	+	+	+	+	+	+	+	+	+	+	−	−
MTC of Co	Concentration of Co (mM)																
	1	1.5	2	2.5	3	3.5	4	4.5	5	5.5	6	6.5	7	7.5	8	8.5	9
	+	+	+	+	+	+	+	+	+	+	+	+	+	−	−	−	−
MMR	Concentration of Ni, Co and Cr (mM) at 1:1:1																
	1	1.5	2	2.5	3	3.5	4	4.5	5	5.5	6	6.5	7	7.5	8	8.5	9
	+	+	+	+	+	+	+	+	−	−	−	−	−	−	−	−	−

**Table 2:** Morphological and biochemical characteristics of isolate AMIC1

Test name	Test results
Morphological tests	
Cell morphology	Rod
Gram’s reaction	G−ve
Motility	Non-motile
Flagella	Absent
Colony characteristics on selective and differential media	
Nutrient agar	White to cream colour colonies
MacConkey’s agar	Pink color colonies
Eosin methylene blue agar	Large mucoid colonies, no metallic sheen
Salmonella Shigella agar	Slight growth, light pink color colonies
TSI agar slant	Yellow colour on butt and slant
Biochemical tests	
Catalase	+
Oxidase	−
Indole	−
VP	+
MR	+
Citrate utilization	+
H_2_S production	−
Carbohydrate fermentation tests	
Arabinose	+
Glucose	+
Inositol	−
Lactose	+
Maltose	+
Mannitol	+
Sucrose	+

*Plus* Positive, *Minus* Negative

**Fig. 2:**
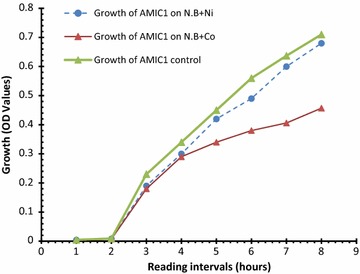
Graph showing effect of Ni and Co vs. control on growth rate of *Klebsiella variicola* strain AMIC1

To observe the effect of Ni and Co on bacterial growth, growth curve experiment was conducted in nutrient broth and results are shown in Fig. 2. Biosorption potential of *K. variicola* AMIC1 was determined through inductively coupled plasma-optical emission spectroscopy (ICP-OES). Percentage reduction in metal concentrations after 24 and 48 h were determined. Results showed that bacteria reduced Ni 49 and 50% whereas reduction of Co was 68.6 and 71% after 24 and 48 h respectively (Table 3).

**Table 3:** Comparison of percentage reduction in Ni and Co by *Klebsiella variicola* strain AMIC1 determined through ICP-OES

Bacterial strain	Metal	24 h (S1)			48 h (S2)			Z value
		Initial	Final	% age	Initial	Final	% age	S1 vs. S2
AMIC1 (*Klebsiella variicola*)	Ni	50	25.5	49	50	25	50	0.20^NS^
	CO	50	15.7	68.6	50	14.5	71	0.22^NS^
Z test	Ni vs. CO	2.07			2.09			

**Fig. 3:**
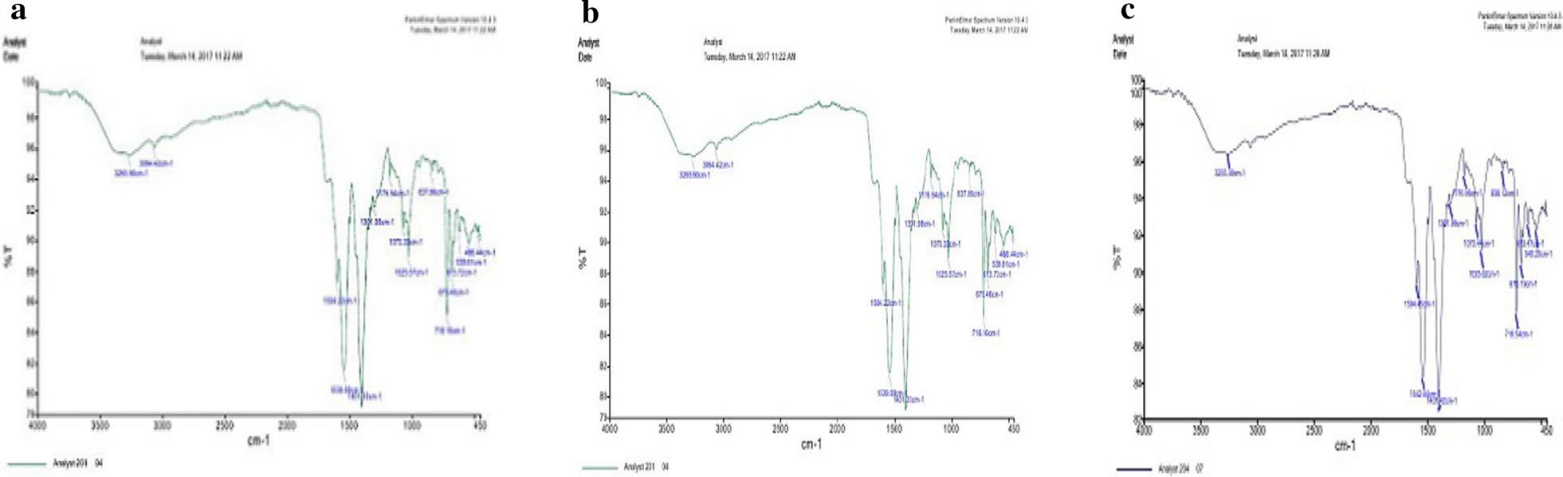
**a** FT-IR spectrum of *Klebsiella variicola* strain AMIC1 without metal loading, **b** with Ni, **c** with Co

To confirm the difference between functional groups in relation to biosorption of metal (Ni and Co), FT-IR analysis was carried out using metal-loaded bacteria in comparison to control (bacteria grown without metal). A change of absorption bands were observed, when compared the FT-IR spectra of control and metal loaded biomass. Figure 3a reflects the spectrum for control and Fig. 3b, c show changes in spectra of biomass after sorption of Ni and Co respectively by *K. variicola* AMIC1. A change in peak at 3500–3200 cm^−1^ region in spectrum of Ni and Co was observed and was considered as the binding of Ni and Co with amino and hydroxyl group. Similarly a change in peak at 1500–1750 cm^−1^ region in spectrum of Ni and Co was observed which indicated the binding of Ni and Co with carboxyl group.

**Fig. 4:**
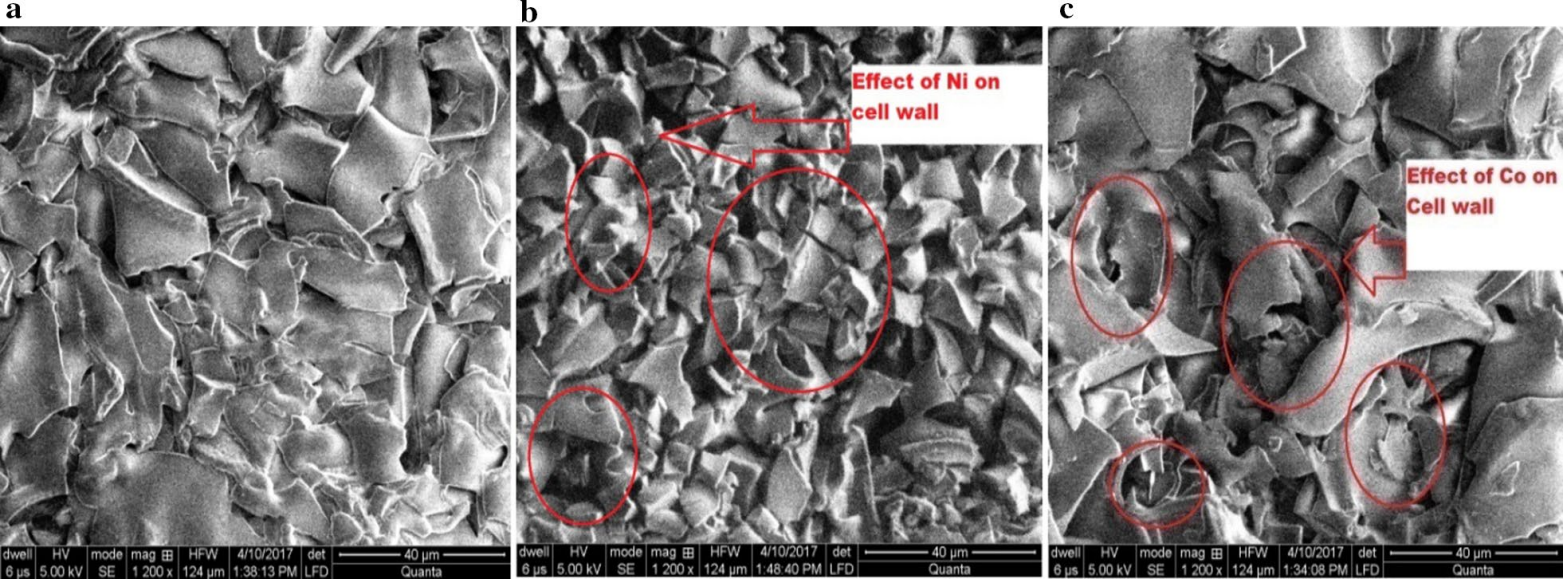
**a** Electron micrograph of *Klebsiella variicola* strain AMIC 1 without metal stress, **b** with Ni, **c** with Co

In SEM, normal electron micrographs of *K. variicola* without metal stress (control) were compared with metal stress to see the surface changes in bacteria due to Ni and Co. The results revealed that both heavy metals showed significant changes in outer membrane of bacteria in terms of roughness of outer membrane, deterioration of normal intact membrane structure, indentation, formation of pores, vaculation etc. as a result of their adsorption with bacterial cell wall and subsequent absorption into the cell. Among two metals, Ni had more effect on bacterial outer membrane than Co (Fig. 4a–c).

## Discussion

Studies showed that presence of metals and other physicochemical parameters play an important role in developing metal tolerance in indigenous bacteria of specific site (Shi et al. 2013). There is an increasing interest and apparently the need of the time for the isolation and identification of some indigenous HMT bacteria and their possible use for the bioremediation of polluted/contaminated areas with heavy metals. Keeping in view the importance of subject, the present study was aimed for the isolation, molecular characterization and evaluation of biosorptive potential of HMT bacteria from textile effluents of Faisalabad, Pakistan.

After initial screening, it was observed that 13 out of total 30 effluent samples exhibited bacterial growth on Nutrient agar incorporated with 0.5 mM of Ni. One sample with code RgrDP_3_ found to have some novel bacterial strains which were able to grow on Nutrient agar incorporated with 08 mM of Ni. It was then screened for tolerance to Co and multi metal resistance (MMR) against Ni, Co and Cr. It was evident from the results that bacteria from RgrDP_3_ was able to tolerate Co up to 7 mM and showed MMR to Ni, Co and Cr (1:1:1) up to 4.5 mM. Isolated bacterial strain which was able to tolerate the maximum concentration of heavy metals isolated from effluent sample RgrDP_3_ was named as AMIC1. After Gram’s staining, it was identified as Gram −ve rods. Molecular characterization of bacterial strain confirmed it as *Klebsiella* sp. showing 99.79% similarity with *K. variicola* DSM 15968^T^ (AJ783916) (Type Strain: CP010523; Accession Number: LT838344).

The results of present study are in agreement with the work of Abd El Hameed et al. (2015) who performed the similar work by isolating the fungi from phosphatic sources and found a negative correlation between isolates and metal concentrations. Similar study was performed by Selvi et al. (2012) for the isolation and characterization of HMT bacteria from tannery effluents and found that all isolates (*Escherichia coli, Bacillus* spp., *Pseudomonas* spp., *Flavobacterium* spp. and *Alcaligenes* spp.) exhibited tolerance to heavy metals in the respective order; Pb > Cu > Zn > Cr > Hg. Similarly, Raja et al. (2006) performed a study for the isolation and characterization of metal tolerant *Pseudomonas aeruginosa* strain and found that isolate showed biosorption potential against all four tested metals (Cd, Cr, Pb and Ni) and the biosorption pattern was found as: Cr (30%) < Cd (50%) < Pb (65%) < Ni (93%). The results of the present study are also in agreement with the work of Alboghobeish et al. (2014) who isolated Nickel resistant bacteria (NiRB) from wastewater polluted with different industrial sources. Similar study was performed by Ahirwar et al. (2016) for the isolation and characterization of heavy metal resistant bacteria from industrial affected soil and found that bacterial strains identified as *Pseudomonas vulgaris, Pseudomonas fluorescence* and *Bacillus cereus* were found to be the most efficient strains in terms of metal resistance.

Then the effect of heavy metals (Ni or Co) was observed on isolated strain. Bacteria were grown without and with metals and the growth patterns were studied. It was evident from the results that metal ions (Ni or Co) significantly (P < 0.05) reduced the rate of growth of bacteria as compared to control group. For the evaluation of biosorptive potential, percentage reduction in metal concentration after 24 and 48 h was determined through ICP-OES. The results showed that *K. variicola* reduced Nickel (Ni) 49 and 50% whereas reduction of Cobalt (Co) was 68.6 and 71% after 24 and 48 h respectively. Results of statistical analyses exhibited non**-**significant difference (P > 0.05) in the metal absorption capacity when incubated for 24 h and 48 h but there was a significant difference (P < 0.05) for biosorption capacity for both metals.

The results of present study are in agreement with the work of Das and Kumari (2016) who found that *Enterobacter* sp. and *Klebsiella* sp. isolated from industrial effluents significantly (P < 0.05) reduced Pb when studied in vitro through ICP-OES. Similar results were reported by Abbas et al. (2014) who found that *Pseudomonas* sp. M3 isolated from wastewater samples were able to reduce 70% Cd from medium. In another study, Abbas et al. (2014) found that *Enterobacter* sp. and *K. pneumonia* sp. isolated from industrial effluents significantly (P < 0.05) reduced Ag. Similar results were documented by Alboghobeish et al. (2014) who found that *K. oxytoca* decreased 83 mg l^−1^ of Ni^2+^ from the medium after 72 h. Similarly, Gawali et al. (2014) evaluated the bioremediation potential of HMT bacteria isolated from industrial wastewater. They found that *E. coli* was able to remove Pb and Cu with removal percentage of 45% and 62% respectively. *P. aeruginosa* was able to remove Cd, Ni and Co with removal percentage of 56, 34 and 53% respectively. While *E. acrogens* was able remove Cd and Cu with removal percentage of 44 and 34% respectively.

FT-IR study was carried out to confirm the difference between functional groups in relation to biosorption of Ni and Co using metal-loaded biomass in comparison to control. The control sample demonstrated the presence of a number of absorption peaks and reflected the complex nature of the biomass. A change of absorption bands were observed, when we compared the FT-IR spectra of control and metal loaded biomass. It was observed that there was a change in peak at 3500–3200 cm^−1^ region in spectrum of Ni and Co and was considered as the binding site of Ni and Co with amino and hydroxyl group. The results of the present study are in agreement with Park et al. (2005) who performed a similar study and described that a peak at 3500–3200 cm^−1^ region is due to the stretching of the N–H bond of amino groups and indicates bonded hydroxyl group. Similarly Kazy et al. (2006) described that the absorption peaks at 2900–3000 cm^−1^ are attributed to the asymmetric stretching of C–H bond of the –CH_2_ groups combined with that of the CH_3_ groups. Pistorius (1995) described that the peaks in the range 1300–1067 cm^−1^ are attributable to the presence of carboxyl and phosphate groups.

In present study, it was evident from the results of SEM that both metals affected bacterial cell wall. Metals adsorbed with the cell wall and created pores in it. It was also observed that effects of Ni was more pronounced than Co. Results are in agreement with the work of Sujatha et al. (2013) who documented that the surface modifications occurred after binding of Ni(II) ions onto the surface of *Trichoderma viride* biomass. In a similar study Chakravarty and Banerjee (2008) reported that there was a clear change (rough cell surface and membrane indentations) in the outer surface of *Acidophilic bacterium* under metal stress condition.

On the basis of overall results of this study, it was concluded that native bacterial strain AMIC1 (*K. variicola*) isolated from industrial effluents of Faisalabad Pakistan possessed considerable heavy metal tolerance ability against Ni and Co and also had significant biosorptive potential against these metals in varying concentration. Therefore it may be a potential candidate to be utilized in future for the development of bioremediation agents to detoxify textile effluents at industrial surroundings within natural environments in Pakistan.

## Abbreviations

Ni: nickel
Co: cobalt
Cr: chromium
HMT: heavy metal tolerance
MTC: maximum tolerable concentration
MMR: multi metal resistance
AMC-1: Abuzar Microbiology 1
FCBP: first fungal culture bank of Pakistan
FT-IR: Fourier transform infrared spectroscopy
ICP-OES: inductively coupled plasma-optical emission spectroscopy
SEM: scanning electron microscopy
